# One-Step Accelerated Synthesis of Conducting Polymer/Silver Composites and Their Catalytic Reduction of Cr(VI) Ions and *p*-Nitrophenol

**DOI:** 10.3390/polym15102366

**Published:** 2023-05-18

**Authors:** Islam M. Minisy, Oumayma Taboubi, Jiřina Hromádková

**Affiliations:** Institute of Macromolecular Chemistry, Czech Academy of Sciences, 162 06 Prague, Czech Republic

**Keywords:** conducting polymers, composites, silver nanoparticles, catalysis, hexavalent chromium ions, *p*-nitrophenol

## Abstract

In this paper, silver nitrate was used as an oxidant to prepare polyaniline, polypyrrole, and poly(3,4-ethylene dioxythiophene)/silver composites through a simultaneous oxidation/reduction process. In addition, *p*-phenylenediamine was added with 1 mole% relative to the concentrations of the monomers to accelerate the polymerization reaction. The prepared conducting polymer/silver composites were characterized by scanning and transmission electron microscopies to study their morphologies; Fourier-transform infrared and Raman spectroscopies to confirm their molecular structures; and thermogravimetric analysis (TGA) to study their thermal stabilities. The silver content in the composites was estimated by energy-dispersive X-ray spectroscopy, ash analysis, and TGA. The conducting polymer/silver composites were utilized for the remediation of water pollutants through catalytic reduction. Hexavalent chromium ions (Cr(VI)) were photocatalytically reduced to trivalent chromium ions, and *p*-nitrophenol was catalytically reduced to *p*-aminophenol. The catalytic reduction reactions were found to follow the first-order kinetic model. Among the prepared composites, polyaniline/silver composite has shown the highest activity for the photocatalytic reduction of Cr(VI) ions with an apparent rate constant of 0.226 min^–1^ and efficiency of 100% within 20 min. Additionally, poly(3,4-ethylene dioxythiophene)/silver composite showed the highest catalytic activity towards the reduction of *p*-nitrophenol with an apparent rate constant of 0.445 min^–1^ and efficiency of 99.8% within 12 min.

## 1. Introduction

**Figure 1:**
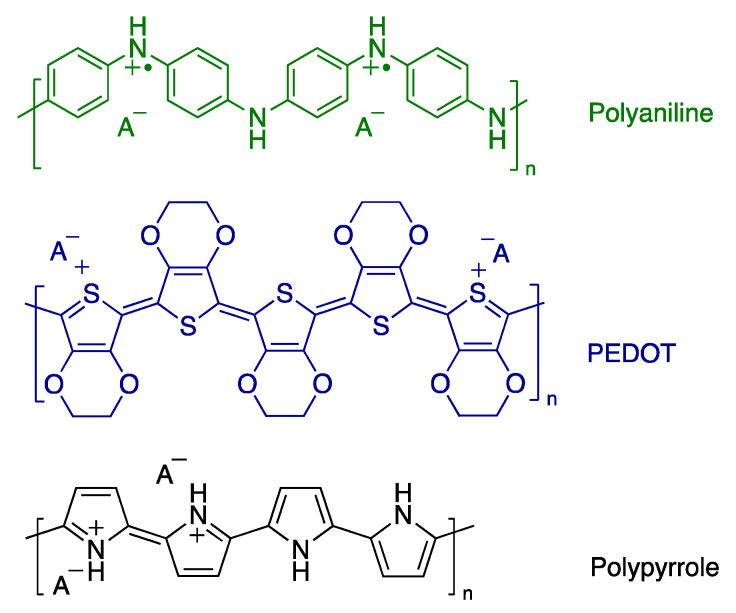
Molecular structures of polyaniline, poly(3,4-ethylene dioxythiophene) (PEDOT), and polypyrrole in their doped forms.

Conducting polymers are a special class of organic polymers that conduct electricity in the region of semiconductors [1]. Polyaniline (PANI), polypyrrole (PPy), and poly(3,4-ethylenedioxythiophene) (PEDOT) are the most studied conducting polymers (Figure 1). They show an excellent environment and thermal stability. They can be easily prepared by the oxidative polymerization of their monomers through either chemical or electrochemical routes [2,3]. Ammonium peroxydisulfate and iron(III) chloride are considered the most frequently used oxidants for the preparation of conducting polymers chemically [4,5,6]. Those oxidants are strong enough to produce conducting polymer powders within minutes with high conductivity (units of S cm^−1^), reasonable yield, and high purity. The successful preparation of conducting polymers with other oxidants such as silver nitrate has also been reported in the literature to produce conducting polymer/silver composites [7,8]; however, it is a time-consuming polymerization process and usually takes several weeks or even months to obtain a reasonable yield [8,9,10].

Composites of PPy/Ag were prepared by UV-induced polymerization of pyrrole in the presence of silver nitrate [11], hydrothermal treatment of pyrrole solution with silver oxide (Ag_2_O) at elevated temperatures [12], or hydrothermal treatment of pyrrole and silver nitrate in the presence of polyvinyl pyrrolidone (PVP) as a stabilizing and capping agent [13]. A few attempts have been made to prepare PEDOT/Ag composites by a one- or two-step approach. Silver particles were deposited with the aid of a reducing agent, such as NaBH_4_ [14,15] or γ-irradiation [16], onto PEDOT. Colloidal PEDOT: PSS/Ag composites were prepared chemically by a one-step method using silver nitrate as the oxidant [17,18], electrochemically [19], or through γ-radiolysis [16]. Similarly, the polymerization rate of aniline using silver nitrate as an oxidant was accelerated by autoclaving [20], UV irradiation [21], or sonication [22]. Interestingly, it was found that the addition of a very low concentration of *p*-phenylenediamine (*p*-PDA)(less than 2 mole% relative to aniline) to the polymerization medium of aniline with silver nitrate significantly enhanced the rate of polymerization and shortened the reaction time to a few hours [23]. Based on this approach, within this study, *p*-PDA is tested to accelerate the polymerization of pyrrole and 3,4-ethylene dioxythiophene as well.

Silver nanoparticles are widely used in catalysis due to the availability of their valance electrons, long-term stability, high surface area, and effective cost [24]. However, silver nanoparticles tend to aggregate in solutions and suffer difficulty in recovering from the reaction medium for reusability purposes [25]. The immobilization of the silver nanoparticles into conducting polymer matrices can provide long-term stability and enhance its electro and catalytic activities [26]. Conducting polymers have high electron mobility, environmental stability, and visible light activity [27]. Moreover, conducting polymers can transfer electrons, facilitating the migration of photogenerated electrons and at the same time hindering the generated charges to recombine [28].

Conducting polymers and their composites have attracted much interest in the field of water treatment to remediate various organic and inorganic contaminants, via adsorption, electrocatalysis, photocatalysis, and chemical degradation approaches [29,30,31]. Such approaches are considered the most effective, eco-friendly, and economical to control water contaminants [32].

Herein, PANI, PPy, and PEDOT composites with silver nanoparticles were one-step prepared in the presence of *p*-PDA and silver nitrate as an oxidant. The composites were characterized by different analytical techniques, e.g., scanning and transmission electron microscopies, energy-dispersive X-ray spectroscopy, FTIR spectroscopy, Raman spectroscopy, thermal gravimetric analysis (TGA), and ash analysis. The conductivity of the prepared composites is not considered in this work, and the main focus is the utilization of conducting polymer/silver composites as heterogeneous catalysts for the remediation of wastewater from Cr(VI) ions as a model of heavy metal pollutants and the reduction of toxic *p*-nitrophenol as a model of persistent organic pollutants.

## 2. Materials and Methods

### 2.1. Chemicals and Reagents

Pyrrole, aniline, 3,4-ethylene dioxythiophene, potassium dichromate (K_2_Cr_2_O_7_), *p*-nitro phenol, *p*-phenylenediamine (*p*-PDA), sodium borohydride (NaBH_4_), and methanesulfonic acid (≥99.0%) were purchased from Sigma-Aldrich (Taufkirchen, Germany). Silver nitrate was obtained from Penta Chemicals Unlimited (Praha, Czech Republic) and formic acid (98%) was obtained from Lach-Ner (Neratovice, Czech Republic). All chemicals were used as received without any further purifications.

### 2.2. Synthesis of Conducting Polymers/Silver Composites

Typically, 0.198 M of a monomer (1.84 g of aniline, 2.82 g of EDOT, 1.33 g of pyrrole) and 2 mM *p*-PDA (0.022 g) were dissolved in 0.2 M methanesulfonic acid solution (50 mL). In another beaker, 0.5 M silver nitrate (8.45 g) was dissolved in 0.2 M methanesulfonic acid solution (50 mL). The previous solutions were mixed and the reactions were left to proceed at room temperature for two weeks before collecting the precipitate by filtration and washed thoroughly with 0.2 M methanesulfonic acid solution and ethanol, then left to dry at room temperature over silica gel.

### 2.3. Characterization

The morphology of the composites was investigated using the Tescan MAIA3 scanning electron microscope and FEI Tecnai G2 Spirit transmission electron microscope. The SEM equipped with an energy-dispersive X-ray spectroscope (EDX) was used to study the elemental composition of the prepared composites. Thermal analysis of the composites was conducted under airflow at the heating rate of 10 °C min^−1^ up to 800 °C with Pyris 1 Thermogravimetric Analyzer (PerkinElmer, Waltham, MA, USA). Fourier-transform infrared (FTIR) spectra of the composite powders were obtained using attenuated total reflection (ATR) infrared spectroscopy with a Thermo Nicolet NEXUS 870 spectrometer equipped with an MCT nitrogen-cooled detector. Raman spectra obtained by excitation line 785 nm were recorded with a Renishaw InVia Reflex Raman microspectrometer (Wotton-under-Edge, UK). The scattered light was analyzed using a spectrograph with holographic gratings of 1200 lines mm^−1^. A research-grade Leica DM LM microscope was used to focus the laser beam. A Peltier-cooled CCD detector (576 × 384 pixels) registered the dispersed light. Ash analysis was used to estimate the silver content in the different conducting polymer composites by burning the composites in the air and calculating the inorganic residual weight.

### 2.4. Photocatalytic Reduction of Hexavalent Chromium Ions

The adsorption/reduction property of Cr(VI) ions by conducting polymer/silver composites was conducted in aqueous solutions. Typically, 6 mg of PANI/Ag, PEDOT/Ag, or PPy/Ag composites were added to 10 mL of 70 mg L^−1^ of Cr(VI) solutions at pH 2 level, under mild stirring (200 rpm) at room temperature. The photocatalytic activities of the composites were evaluated for the reduction of toxic Cr(VI) to Cr(III) under irradiation of warm white LED light (660 lm). An amount of 0.5 M formic acid (HCOOH) as a hole scavenger was used to inhibit electron–hole recombination and enhance the photocatalytic reduction of Cr(VI) ions [33]. The reduction of Cr(VI) ions was followed by measuring the UV–Visible absorbance over time using a Thermo Scientific UV–Visible spectrophotometer (Evolution 220).

### 2.5. Catalytic Reduction of p-Nitrophenol

In a well-stoppered quartz cuvette, conducting polymer/silver composite (1 mg) was added to 2 mL of an alkaline *p*-nitrophenol solution (0.1 mM), and the UV–Visible absorption spectra were recorded. After that, 1 mL of freshly prepared NaBH_4_ solution (0.2 M) was added to the previous solution, and the progress of the catalytic reduction reaction at room temperature was followed by measuring the UV–Visible absorption spectra over time.

The catalytic efficiency was normalized by *C_t_/C*_0_, where *C_t_* is the unreacted Cr(VI) or *p*-nitrophenol concentrations at time *t* and *C*_0_ is their initial concentrations. The results were treated as first-order kinetics (Equation (1)) by plotting ln(*C_t_*/*C*_0_) versus *t*. The apparent rate constant (*k_app_*) is calculated from the slope of fitting lines.

(1) $$\ln\left(C_{t}/C_{0}\right)=–k_{app}t$$

## 3. Results and Discussion

### 3.1. Oxidation of the Monomers

Conducting polymers are prepared chemically by the oxidative polymerization of their acidified monomer solutions. However, the standard reduction potential of Ag^+^ ions (+0.80 V) is much lower than the peroxydisulfate reduction potential (+2.01 V), it is slightly higher than the reduction potential of iron(III) chloride (+0.77 V) [34]. It is thus not surprising that silver ions have similar oxidation capabilities to form conducting polymers. Unlike the fast polymerization rate, when using ammonium peroxydisulfate or iron(III) oxidants, polymerization with silver nitrate as an oxidant is very slow and characterized by an induction period over weeks [9]. To accelerate the rate of polymerization, 2 mM of *p*-PDA (1 mol% relative to monomer concentration) was added to the polymerization media. This reduced the time of polymerization from months to a few days. The oxidative polymerization of pyrrole, aniline, and 3,4-ethylene dioxythiophene with silver nitrate leads to the simultaneous oxidation of monomers into polymers and reduction of Ag^+^ ions to Ag^0^ metal and hence forms conducting polymer/silver composites. The simultaneous oxidation–reduction of monomer and silver ions facilitates the incorporation of silver metal particles within the conducting polymer matrix via adsorption or electrostatic interaction [35,36,37].

In the case of PANI preparation, the presence of methanesulfonic acid (0.2 M) as a dopant and *p*-PDA was crucial for the reaction to proceed. The addition of methanesulfonic acid to the polymerization medium drastically increases the yield and prevents the formation of a silver mirror on the wall of the polymerization beaker. In the case of PEDOT preparation, the yield was enhanced, and the reaction rate was faster in the presence of *p*-PDA (Table 1). PPy was obtained with the same yield in the presence or absence of *p*-PDA, counting on the long polymerization time in the absence of *p*-PDA. This observation is in line with the previously published findings [23]. It is proposed that *p*-PDA participates in the formation of initiation centers that start the propagation of the conducting polymer chains in the media of sufficient acidity [38].

### 3.2. Morphology

**Figure 2:**
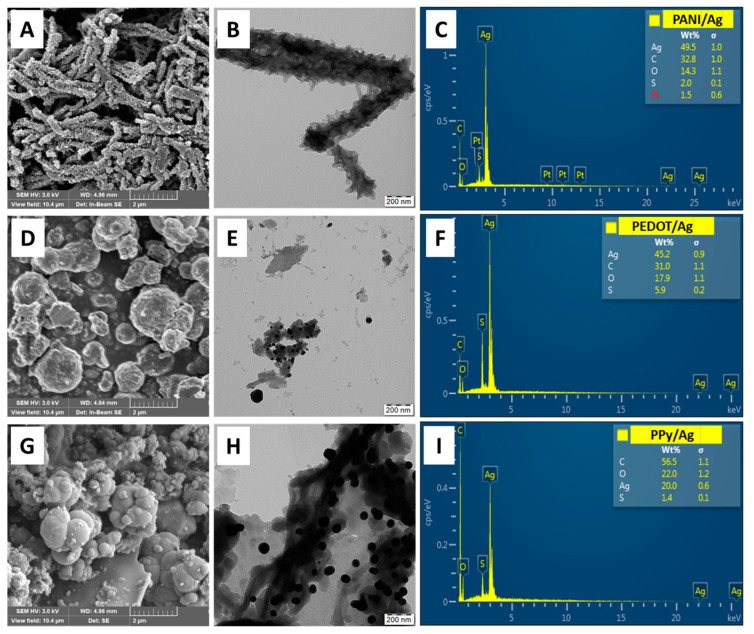
SEM and TEM micrographs and the corresponding SEM/EDX analysis of (**A**–**C**) polyaniline/silver, (**D**–**F**) PEDOT/silver, and (**G**–**I**) polypyrrole/silver composites prepared in the presence of *p*-phenylenediamine and methanesulfonic acid.

The morphological study of the conducting polymers/silver composites prepared in the presence of *p*-PDA and methanesulfonic acid shows the formation of discrete silver particles embedded into the conducting polymer matrix (Figure 2). PPy/Ag and PEDOT/Ag composites have typical globular structures similar to neat PPy and PEDOT prepared with iron(III) chloride and ammonium peroxydisulfate oxidants. PANI/Ag composite shows a fibrillar-like morphology with dense spikey surfaces. A similar PANI fibrillated morphology was obtained in the case of aniline polymerization with silver nitrate as an oxidant in nitric acid and the absence of *p*-PDA [9]. The morphological structures of the conducting polymer/silver composites are mainly composed of the conducting polymer, and the volume fraction of silver content is low due to the high density of silver compared to the conducting polymer matrices. As appeared from the TEM analysis, silver particles are included within conducting polymer matrix, preventing them from aggregation and possible leaching.

### 3.3. Thermogravimetric Analysis

**Figure 3:**
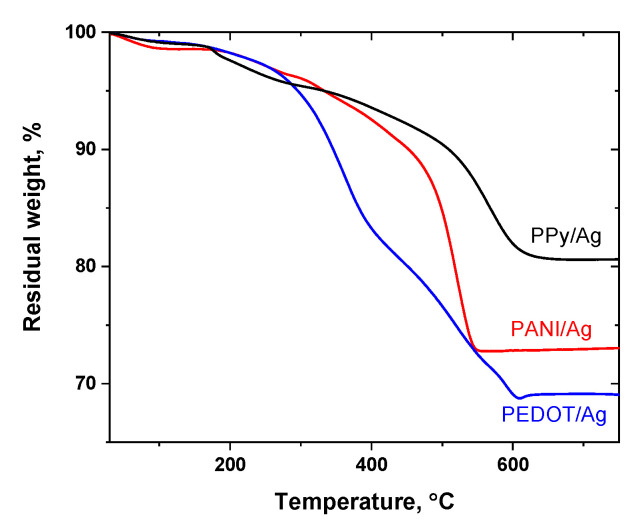
Thermogravimetric analysis of polyaniline/silver, PEDOT/silver, and polypyrrole/silver composites prepared in the presence of *p*-phenylenediamine and methanesulfonic acid.

The thermal stability of the conducting polymer/silver composites prepared in the presence of *p*-PDA and methanesulfonic acid as a dopant was studied by thermogravimetric analysis under airflow conditions (Figure 3). About 10 mg of the different composite powders were gradually heated up to 800 °C. All the composites show a gradual weight loss associated with absorbed water loss due to the hygroscopic nature of conducting polymers, and the dedoping process by the evaporation of methanesulfonic acid dopant (the boiling point of methanesulfonic acid is 167 °C). Then, the degradation of polymeric backbones of the conducting polymers and full decomposition of the organic content takes place at higher temperatures. Among the studied composites, PPy/Ag composite showed the highest thermal stability, and the organic content of the composite was fully decomposed at 665 °C. PEDOT/Ag composite polymeric content was fully decomposed at 605 °C, and PANI/Ag is decomposed at around 550 °C. The residual weights represent the silver content (Table 2).

### 3.4. Silver Content

The silver content in the composites was determined by energy dispersive spectroscopy (SEM/EDX), TGA, and from the residual weight of ash after burning the composites in the air. The good agreement between the results obtained from the TGA and ash analysis indicates the homogeneity of the composites to a certain extent. The silver content calculated by SEM/EDX spectroscopy is different, SEM/EDX is a semiquantitative surface analysis technique. Silver content may differ on the surface and bulk of the composites. Ash analysis results are more representative of both surface and bulk silver content (Table 2).

The ratio of the silver mass in the composite (calculated from ash analysis) to the mass of silver entered into the reaction as silver nitrate is presented as *r* in Table 2. Considering the full conversion of monomer to the polymer and neglecting the weight increase by the dopant incorporation, the *r* values (Table 2) show that the content of silver is very close to the stoichiometric calculations and confirms the full conversion of silver ions to metallic silver. The obtained results are in accordance with the results reported before; the content of silver was close to the theoretical calculations [23].

### 3.5. FTIR and Raman Spectroscopy

**Figure 4:**
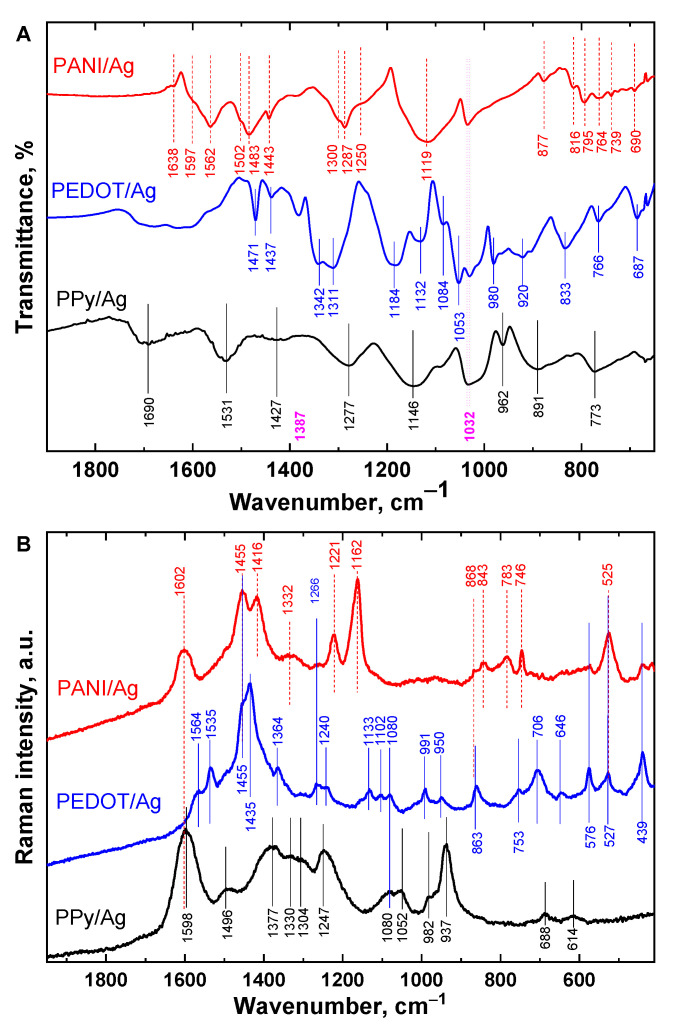
(**A**) FTIR and (**B**) Raman spectra of polyaniline/silver, PEDOT/silver, and polypyrrole/silver composites prepared in the presence of *p*-phenylenediamine and methanesulfonic acid.

The molecular structures of PANI/Ag, PEDOT/Ag, and PPy/Ag composites were studied by vibrational spectroscopy; FTIR and Raman spectroscopies (Figure 4).

As can be seen in Figure 4A, PPy peaks are located around 1531 and 1427 cm^−1^, which correspond to the C=C and C–N stretching vibration in the pyrrole ring, respectively [39]. The band at 1690 cm^−1^ can be related to the overoxidation of PPy. The band at 1277 cm^−1^ corresponds to C−N in-plane bending. The breathing vibration of the pyrrole ring is given at 1146 cm^−1^ and the peak at 962 cm^−1^ is attributed to the C–H out-of-plane deformation vibrations. Indeed, the characteristic bands are assigned to the vibration of typical PPy aromatic rings.

The FTIR spectrum of the PEDOT/Ag shows the same characteristic bands of PEDOT prepared with iron(III) chloride [40]. Bands corresponding to C=C and C–C in the thiophene ring are observed at 1471 and 1342 cm^−1^. The stretching vibrations of the C–S–C bond in the thiophene ring can be seen at 920, 833, 766, and 687 cm^−1^. The vibrations at 1184, 1132, 1084, and 1053 cm^−1^ are assigned to C–O–C stretching in the ethylenedioxy group [40].

On the other hand, the spectrum of the PANI/Ag composite proves the presence of aniline oligomers with PANI chains. The main characteristic peaks of PANI are assigned as follows: The bands at 1597, 1562, and 1483 cm^−1^ correspond to quinonoid and benzenoid ring-stretching deformations. The bands 1287 and 1250 cm^−1^ are assigned to the C−N stretching of secondary aromatic amine and C–N**^•^**^+^ stretching in the polaron lattice of PANI, respectively. The small sharp peak at 1443 cm^−1^ belongs to the skeletal C=C stretching vibration of the substituted aromatic ring which is typical of aniline oligomers [41]. In addition, the bands at 816 (C–H out-of-plane bending of two adjacent hydrogen atoms on a 1,4-disubstituted benzene ring), 764, 739, 690 cm^−1^ (C–H out-of-plane bending and out-of-plane ring deformations of a mono-substituted phenylene ring), and 1638 cm^−1^ may be due to the presence of aniline oligomers [41]. The bands at 1387 cm^−1^ and 1032 cm^−1^ belong to nitrate and sulfonate anions, respectively. PEDOT and PANI were found to be doped with nitrate and methanesulfonic acid, while the PPy spectrum shows only methanesulfonic acid as a dopant.

Raman spectroscopy is a complementary technique to FTIR and can provide information about the protonation and oxidation states of PANI/Ag, PEDOT/Ag, and PPy/Ag composites (Figure 4B). The Raman spectrum of the PPy/Ag composite shows its characteristic peaks as identical to the neat conventional PPy reported earlier in the literature [42]. Through the relative intensities of the two peaks, 1377 cm^−1^ (ring stretching in bipolaron structure) and 1330 cm^−1^ (ring stretching in polaron structure), and of the two peaks 937 cm^−1^ (ring deformation in bipolaron structure) and 982 cm^−1^ (ring deformation in polaron structure), PPy chains exist in its protonation form.

The PEDOT/Ag composite Raman spectrum shows a series of bands at 1564 cm^−1^ (C–C asymmetric stretching), 1535 cm^−1^ (C–C asymmetric stretching), 1435 cm^−1^ (C–C symmetric stretching), 1364 cm^−1^ (C–C symmetric stretching), 1266 cm^−1^ (C–C symmetric stretching inter-ring), 1133 cm^−1^ (C–H bending), 1102 cm^−1^ (C–O–C bond deformation), 991 cm^−1^ (oxyethylene ring deformation), 753 cm^−1^ (C–S–C bond deformation), 706 cm^−1^ (C–S–C bond deformation), and 439 cm^−1^ (C–O–C deformation), which proves full oxidation of EDOT to PEDOT in the composite [43,44].

The Raman spectrum of PANI/Ag composite shows the band at 1602 cm^−1^ of benzenoid stretching vibration, the band at 1455 cm^−1^ with a small shoulder at 1497 cm^−1^ are assigned to C=N stretching vibration in quinonoid units [38], the band at 1416 cm^−1^ is assigned to the phenazine-like segment vibration. The C∼N**^•^**^+^ stretching vibration of delocalized polaronic structures is given at 1332 cm^−1^. The sharp peak at 1162 cm^−1^ is attributed to C–H bending vibration of the semi-quinonoid rings.

For all the prepared composites, the addition of *p*-PDA has shown no detectable effect on the chemical structures of the conducting polymers.

### 3.6. Photocatalytic Reduction of Hexavalent Chromium Ions

**Figure 5:**
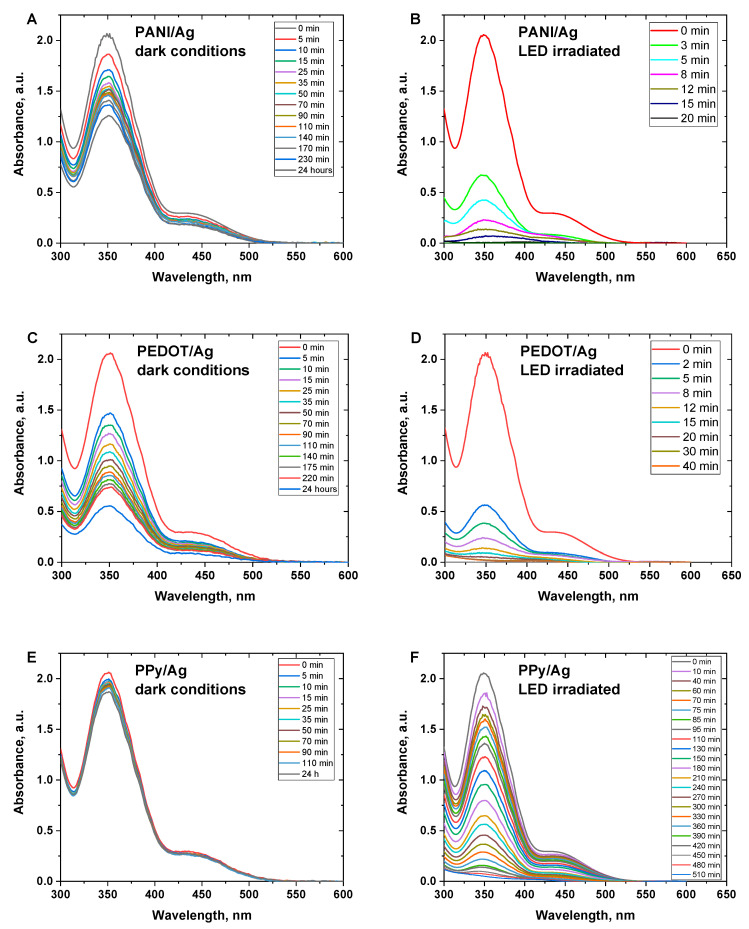
Time-dependent UV–Visible spectra of Cr(VI) ions solution (*C*_0_ = 70 mgL^–1^) in the presence of conducting polymer/silver composites in (**A**,**C**,**E**) dark conditions, and (**B**,**D**,**F**) under white LED light irradiated conditions.

Hexavalent chromium ions (Cr(VI)) are extremely toxic and identified as a human carcinogen. PANI, PPy, and their composites have been widely used for the adsorptive elimination of Cr(VI) ions from aqueous solutions under acidic conditions [45,46,47]. Before evaluating the photocatalytic reduction of Cr(VI) under white LED light irradiation, Cr(VI) ions adsorption on the surface of the composites was performed in dark conditions. Figure 5A,C,E shows the time-dependent Cr(VI) spectra when Cr(VI) ions are adsorbed onto the various conducting polymer/silver composites. The characteristic peak of Cr(VI) at 350 nm was found to decrease gradually with time to a specific extent. Among the various composites, PEDOT/Ag composite has the highest adsorption capacity with a removal efficiency of ∼73%, PANI/Ag composite showed a removal efficiency of ∼41%, and PPy/Ag composite showed the lowest removal efficiency of ∼10% only, after 24 h.

The photocatalytic activity of the composites was evaluated under white LED light irradiation. Figure 5B,D,F shows the time dependence of photocatalytic reduction of Cr(VI) under light irradiation in the presence of various conducting polymer/silver composites.

The characteristic peak of Cr(VI) at 350 nm was found to considerably decrease within a much shorter time compared to the corresponding dark conditions. Results reveal that PANI/Ag composite has the highest activity towards the full reduction of the toxic Cr(VI) ions to the less toxic Cr(III) ions. PEDOT/Ag composite showed high activity as well for the reduction of Cr(VI) ions. PPy/Ag composite showed the lowest rate of catalytic reduction, and the full conversion of Cr(VI) to Cr(III) was achieved after a relatively long time of ∼9 h. PEDOT/Ag composite showed much higher activity compared to the recently reported PEDOT/TiO_2_ composite, which had a maximum efficiency of only 63% within 60 min [48].

**Figure 6:**
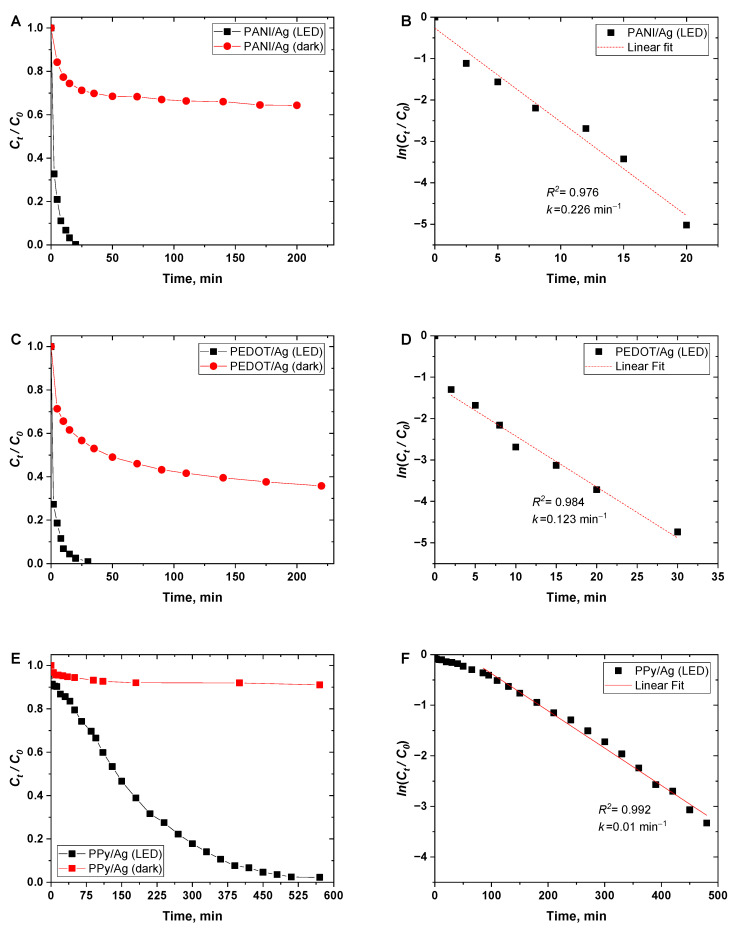
(**A**,**C**,**E**) Variation in normalized (*C_t_/C*_0_) concentration of Cr(VI) (*C*_0_ = 70 mgL^–1^) ions in dark and under white LED light irradiation conditions as a function of time, and (**B**,**D**,**F**) ln(*C_t_/C*_0_) as a function of time, when using polyaniline/silver, PEDOT/silver, and polypyrrole/silver composites as photocatalysts.

Figure 6A,C,E shows the big differences in the Cr(VI) removal efficiency between the dark and LED light-irradiated conditions. The removal efficiency of 100% of Cr(VI) was obtained in the case of PANI/Ag and PEDOT/Ag composites within 20 and 40 min, respectively, whereas PPy/Ag composites needed 8.5 h for an efficiency of 98.5%. Additionally, it was found that the catalytic reduction of Cr(VI) has a good agreement with the first-order kinetic model (Figure 6B,D,F) with apparent rate constants of 0.226, 0.123, and 0.01 min^–1^ for PANI/Ag and PEDOT/Ag, and PPy/Ag composites, respectively.

**Figure 7:**
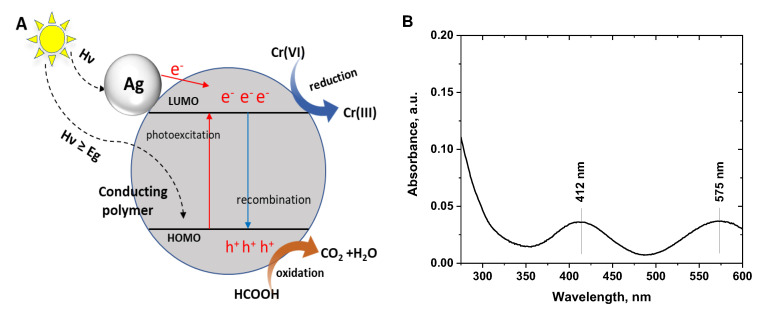
(**A**) Proposed photocatalytic reduction mechanism of hexavalent chromium (Cr(VI)) ions by conducting polymer/silver composites, (**B**) Spectrum of the Cr(VI) solution (70 mg/L) after photocatalytic reduction with polyaniline/silver composite.

The possible mechanism of the photocatalytic reduction of Cr(VI) to Cr(III) is depicted in Figure 7A. Photoexcitation of the irradiated conducting polymer/silver composites generates electrons and holes. Silver nanoparticles can absorb light in visible and UV regions [49], and the photoexcited electrons may migrate from the conduction band in silver to the LUMO level of conducting polymers or directly reduce Cr(VI) to Cr(III) ions. The photogenerated electrons in the conducting polymer migrate from HOMO to LUMO levels and to Cr(VI) ions. Conducting polymers show good separation efficiency of photogenerated electron–hole pairs, in addition, formic acid was added to the reaction medium as a hole scavenger to hinder electron–hole recombination and accelerate the photocatalytic reduction of Cr(VI) ions [33,50].

The photocatalytic reduction of Cr(VI) ions to Cr(III) ions was confirmed from the UV–Visible spectra of the solutions (Figure 7B) that show the weak characteristic peaks of Cr(III) at 412 and 575 nm [51]. Additionally, after the photocatalytic reduction of Cr(VI) solutions with the conducting polymer/silver composites, the solutions turn into a grey/green color when adding concentrated NaOH solution, which confirms the formation of Cr(III) through the reduction.

### 3.7. Catalytic Reduction of p-Nitrophenol

*p*-nitrophenol is a toxic and refractory pollutant that shows high chemical and biological stability against degradation [52]. The best strategy for the remediation of *p*-nitrophenol is to convert it to a less toxic form by reduction to *p*-aminophenol [53]. The reduction reaction of *p*-nitrophenol to *p*-aminophenol with NaBH_4_ is generally employed as a model reaction for the investigation of the catalytic activity of noble metal composites under ambient conditions.

**Figure 8:**
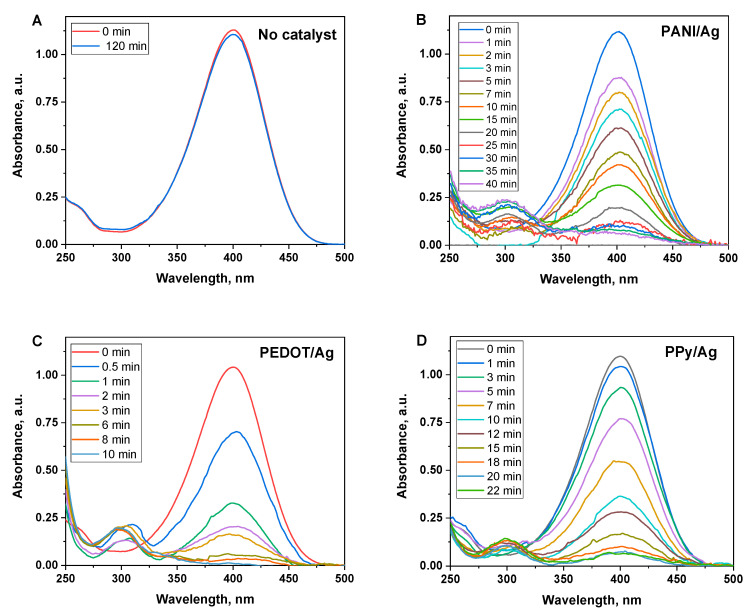
Time-dependent UV–Visible spectra of *p*-nitrophenol solutions after adding NaBH_4_ at the conditions of (**A**) the absence of the catalyst, and the presence of (**B**) polyaniline/silver, (**C**) PEDOT/silver, and (**D**) polypyrrole/silver composites as catalysts.

However, NaBH_4_ can spontaneously reduce *p*-nitrophenol into *p*-aminophenol, the reaction is very slow without a catalyst (Figure 8A) [54]. Herein we investigate the catalytic activity of PANI/Ag, PEDOT/Ag, and PPy/Ag composites, which have been demonstrated as efficient photocatalysts in the previous section. An excess concentration of NaBH_4_ was used to exclude its influence on the reaction rate, and the reduction reaction is treated in terms of first-order kinetics.

The reduction reaction was followed by UV–Visible spectroscopy, which shows the disappearance of the characteristic peak of *p*-nitrophenolate ions at 400 nm and the appearance of a new peak at 290 nm that corresponds to the *p*-aminophenolate ions [55]. As the reaction proceeds, the peak at 400 nm gradually reduces as a function of time, which is associated with the reduction of *p*-nitrophenol (Figure 8). Figure 8B shows that *p*-nitrophenol was completely converted into *p*-aminophenol in the presence of PEDOT/Ag composite as a catalyst within 10 min. The big fluctuation in the UV–Visible baseline is due to the release of H_2_ gas bubbles formed by the reducing agent (NaBH_4_).

**Figure 9:**
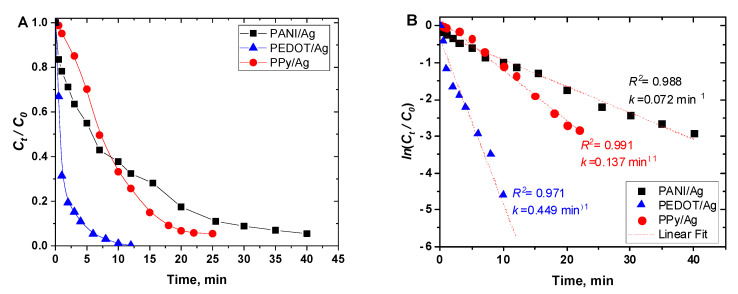
(**A**) Reduction of *p*-nitrophenol to *p*-aminophenol (*C_t_/C_0_*), and (**B**) ln(*C_t_/C_0_*), using polyaniline/silver, PEDOT/silver, and polypyrrole/silver catalysts, as a function of time.

Figure 9A shows the change in the normalized concentration (*C_t_/C*_0_) of *p*-nitrophenol versus time in the presence of PANI/Ag, PEDOT/Ag, and PPy/Ag composites as catalysts. PEDOT/Ag composite has the highest catalytic activity, which may be due to the well-dispersed silver nanoparticles within the PEDOT matrix.

The relation between ln(*C_t_*/*C*_0_) and reaction time was found to be linear for all the catalysts (Figure 9B), which indicates that the reaction follows pseudo-first-order kinetics with respect to the concentration of *p*-nitrophenol only as NaBH_4_ was added in excess concentration. The apparent rate constant (*k_app_*) was calculated from the slope of the plots of ln(*C_t_*/*C*_0_) vs. time (Figure 9B).

**Figure 10:**
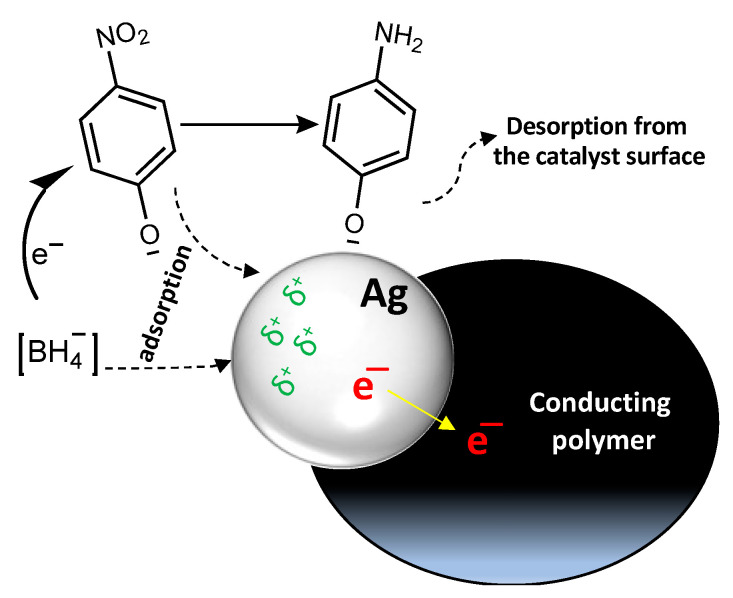
Catalytic reduction mechanism of *p*-nitrophenol to *p*-aminophenol by conducting polymer/silver composites.

Conducting polymer/silver composites serve as an electron relay for the oxidant and reductant. The interfacial interactions between the silver and conducting polymer promote the migration of electrons from silver to the conducting polymer, this makes the silver particles electron deficient (Figure 10). The negatively charged *p*-nitrophenolate and BH_4_^−^ ions preferably interact with silver particles; therefore, electrons and reactive hydrogen are effectively transferred through the catalyst [56]. BH_4_^−^ donates electrons to the electron-deficient catalytic canter and activates hydrogen atoms through the B–H bond fissure. The thermodynamically unstable hydrogen atoms will react with the *p*-nitrophenolate through a hydrogenation process and reduce it to *p*-aminophenolate. When the reaction finishes, the resultant products split from the surface of the catalyst to the solution, and the catalyst surface is regenerated [57].

The catalytic activity is highly dependent on the size of silver particles, available active sites for the adsorption of the reactants, and charge transfer efficiency [56]. From the TEM images (Figure 2), it is clear that PEDOT/Ag composite has well-distributed silver nanoparticles with a smaller particle size compared to PPy/Ag and PANI/Ag composites.

## 4. Conclusions

Hybrid composites of conducting polymers and silver metal nanoparticles were easily prepared by a one-step polymerization of aniline, pyrrole, and 3,4-ethylenedioxythiophene with silver nitrate as the oxidant at room temperature. The rate of the polymerization and the yield of the prepared composites have been significantly enhanced by the addition of *p*-PDA, which alters the polymerization mechanism. The composites of PANI/Ag, PPy/Ag, and PEDOT/Ag, which combine simple preparation, high stability, and relatively low-cost were tested for the remediation of widely spread water recalcitrant contaminants. The composites showed high activity towards the photocatalytic reduction of Cr(VI) ions and *p*-nitrophenol. The comparative study of the catalytic activity of the prepared composites revealed that PANI/Ag composite has the highest activity for the photocatalytic reduction of Cr(VI) ions and PPy/Ag composite has the lowest activity. In addition, PEDOT/Ag composite showed the best activity for the reduction of *p*-nitrophenol, while PANI/Ag was the poorest. All the catalytic reactions were found to follow the first-order kinetics.

## Figures and Tables

**Table 1:** Comparison of the yield of the conducting polymer/silver composites in the presence and absence of *p*-PDA.

Composite	Yield, g *	
	Absence of *p*-PDA	Presence of *p*-PDA
PANI/Ag	No yield	7.03
PEDOT/Ag	4.58	7.66
PPy/Ag	6.89	6.88

* two months were allocated for collecting the yield of the composites in the absence of *p*-PDA, and one week for the composites prepared in the presence of *p*-PDA.

**Table 2:** Silver content in the composites prepared in the presence of *p*-PDA, obtained from the SEM/EDX, TGA, and ash analysis.

Composite	Ag, wt%			*r*
	SEM/EDX	TGA	Ash Analysis	
PANI/Ag	49.5	72.5	71.1	0.96
PEDOT/Ag	45.2	69.1	68.5	1.05
PPy/Ag	20.0	80.3	77.4	0.97

## Data Availability

Data are available upon request.
